# Erratum: Ismail, I.T.; et al. Inborn Errors of Metabolism in the Era of Untargeted Metabolomics and Lipidomics. *Metabolites* 2019, *9*, 242

**DOI:** 10.3390/metabo10010025

**Published:** 2020-01-06

**Authors:** Israa T. Ismail, Megan R. Showalter, Oliver Fiehn

**Affiliations:** 1 National Liver Institute, Menoufia University, Shebeen El Kom 55955, Egypt; israataher2015@gmail.com; 2 NIH West Coast Metabolomics Center, University of California Davis, Davis, CA 95616, USA; mshowalter@ucdavis.edu

The authors wish to make the following correction to this paper [1].

Reference [2] was cited in connection with concept Figure 5, but must be also cited in our Figure 5 legend. Figure 1 of Reference [2] was re-used as part of our concept Figure 5.

The correct Figure 5 legend should be:

**Figure 5.** Improvement of IEM diagnosis tests using untargeted metabolomics. Re-use of Figure 1 from [176], with permission from the publisher.

The authors would like to apologize for any inconvenience caused to the readers by these changes.
